# Cellular toxicity pathways of inorganic and methyl mercury in the green microalga *Chlamydomonas reinhardtii*

**DOI:** 10.1038/s41598-017-08515-8

**Published:** 2017-08-14

**Authors:** Rebecca Beauvais-Flück, Vera I. Slaveykova, Claudia Cosio

**Affiliations:** Environmental Biogeochemistry and Ecotoxicology, Department F.-A. Forel for environmental and aquatic sciences, Earth and Environmental Sciences, Faculty of Sciences, University of Geneva, 66, boulevard Carl-Vogt, 1211 Geneva 4, Switzerland

## Abstract

Contamination by mercury (Hg) is a worldwide concern because of Hg toxicity and biomagnification in aquatic food webs. Nevertheless, bioavailability and cellular toxicity pathways of inorganic (IHg) and methyl-Hg (MeHg) remain poorly understood. We analyzed the uptake, transcriptomic, and physiological responses in the microalga *Chlamydomonas reinhardtii* exposed to IHg or MeHg. Bioavailability of MeHg was up to 27× higher than for IHg. Genes involved in cell processes, energy metabolism and transport were dysregulated by both Hg species. Physiological analysis revealed an impact on photosynthesis and reduction–oxidation reaction metabolism. Nevertheless, MeHg dysregulated a larger number of genes and with a stronger fold-change than IHg at equivalent intracellular concentration. Analysis of the perturbations of the cell’s functions helped to derive a detailed mechanistic understanding of differences in cellular handling of IHg and MeHg resulting in MeHg having a stronger impact. This knowledge is central for the prediction of impact of toxicants on organisms.

## Introduction

Widespread mercury (Hg) contamination of aquatic environment leads to accumulation of high levels of Hg in organisms and can have a detrimental effect on biota and humans^1–5^. Exposure of primary producers such as phytoplankton to low concentrations of inorganic Hg (Hg^2+^, IHg) and methyl-Hg (CH_3_Hg^+^, MeHg) can threaten the aquatic systems functioning^6, 7^. IHg and MeHg exist in different forms, i.e. free uncomplexed ion, bound to natural ligands, particles and colloids, each with different reactivity, biological availability and toxicity in biota^7, 8^. There is substantial evidence that exposure to both IHg and MeHg induces general toxic effects in primary producers including reduction of growth and photosynthesis as well as oxidative stress (see refs 6 and 7 for review). Nevertheless, differences between the fate and the toxicity of IHg and MeHg in organisms have been observed^9–11^. Field studies have shown higher uptake of MeHg than IHg, as well as exclusive biomagnification of MeHg in the food chain^9^. Exposure to IHg increased the lifetime of chlorophyll fluorescence by blocking the photosynthetic electron chain in the marine diatom *Thalassiosira weissflogii* while MeHg had no significant effect^10^. IHg affected the plasma membrane integrity whereas MeHg disturbs organelle metabolism in the cytoplasm, subsequently affecting membrane integrity^11^. It was recently shown that sub-nanomolar concentrations of MeHg significantly altered the energy metabolism (photosynthesis and sugars) and the antioxidant activity of the green microalga *Chlamydomonas reinhardtii* ^12^. However, most of these evidences were obtained in studies conducted at 10^3^ to 10^6^ higher Hg concentrations than those typically found in natural water and so limiting their environmental relevance. Furthermore, the above reported Hg–induced effects are classically assessed by examining the growth, or physiology perturbation of organism but these parameters fall short of revealing subtle effects occurring prior changes at the organism level and tolerance responses that allow the organism to adapt and recover at environmental concentrations. Consequently, little is known about both the impact of IHg and MeHg on microalgae at low environmental Hg concentrations and the possible cellular toxicity pathways of IHg and MeHg in aquatic primary producers.

In this context, transcriptomics (e.g. RNA-Sequencing; RNA-Seq) is considered to be a powerful tool for early detection of adverse outcome pathways as well as tolerance response^13^, notably when analyzed concomitantly with other endpoints^14^. By following the transcriptomic and physiological responses in parallel with cellular Hg concentrations, the comparative study presented here aims to explore the influence of a range of IHg and MeHg concentrations below and above the European environmental quality standard for Hg (2.5·10^−9^ M)^15^ on the green alga *C. reinhardtii*. The following key questions were addressed: (i) What are the cellular toxicity pathways in *C. reinhardtii* exposed to IHg and MeHg? (ii) How similar or different are they for IHg and MeHg? More in details, the effects of IHg and MeHg on the microalga *C. reinhardtii* were assessed by studying the dysregulation of gene expression, the generation of intracellular reactive oxygen species (ROS), and the photosynthesis efficiency at the physiological level, and by exploring the link with the intracellular concentrations of IHg and MeHg, used as a measure of Hg species bioavailability.

## Results and Discussion

### IHg and MeHg bioavailability to *C. reinhardtii*

Speciation modelling of IHg and MeHg in the exposure medium revealed that neutral hydroxo- (-OH) and chloro- (-Cl) complexes were predominant for both IHg and MeHg (Figure S1). The cation CH_3_Hg^+^ represented 0.3% of the total MeHg in the medium. The most abundant IHg cation, HgCl^+^ reached 6·10^−3^%, while the free ion Hg^2+^ concentrations represented only 1·10^−6^% of the total IHg (Figure S1). These results suggest that the processes at medium-alga interface determining bioavailability may involve different predominant species in IHg and MeHg exposure^7, 8^. Nevertheless, although multicomponent thermodynamic equilibrium speciation modeling is widely used and highly informative, it also involves significant uncertainties due to the model assumptions and available stability constants^16^.

**Figure 1:**
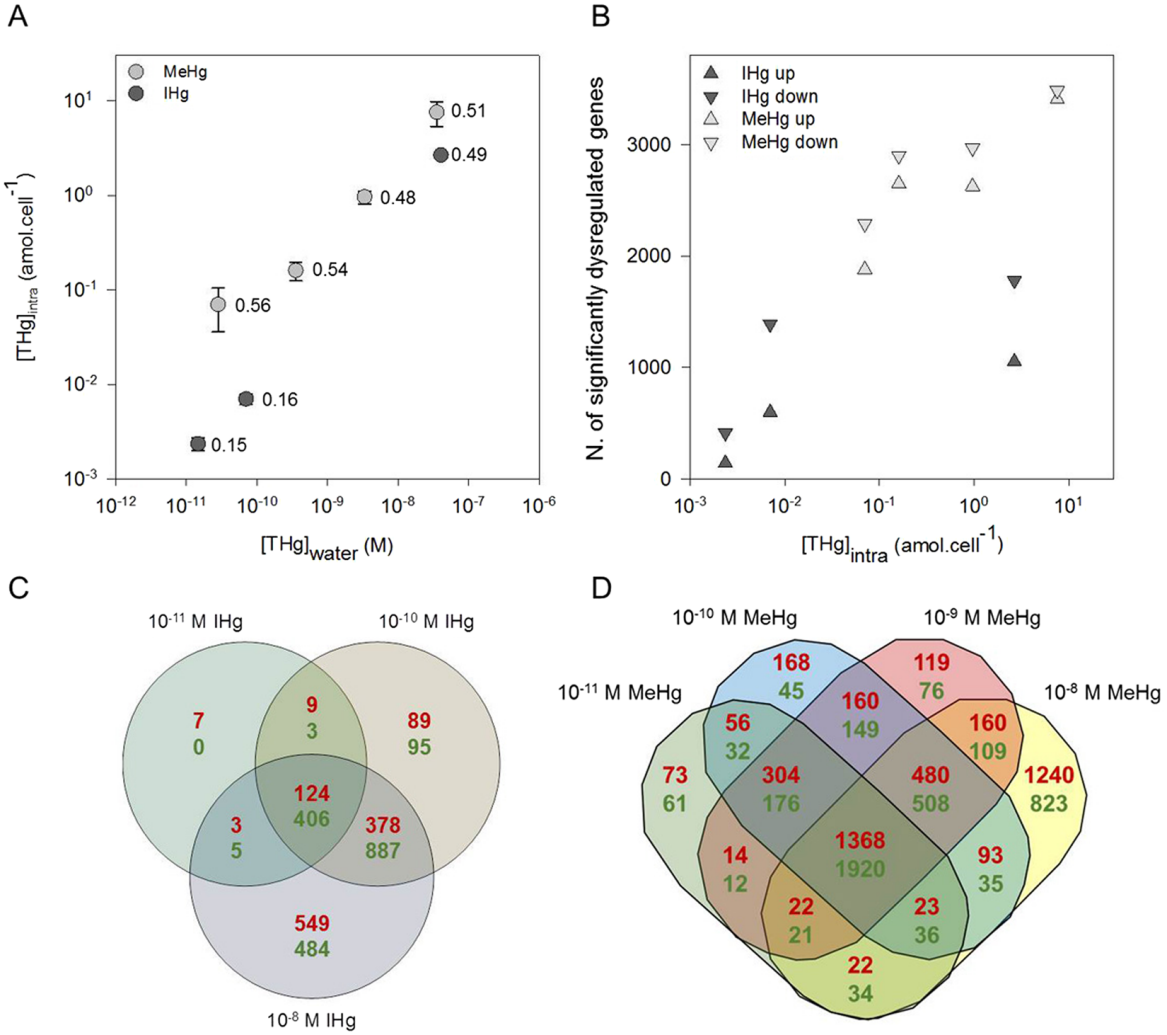
Intracellular total Hg concentrations ([THg]_intra_, mean ± sd, n = 3) in *C. reinhardtii* exposed to IHg or MeHg, as a function of initial IHg or MeHg exposure concentration expressed as THg ([THg]_water_). Values indicate ratio of [THg]_intra_ to [THg]_intra+ads_, lines show linear regressions of log_10_ values (**A**). Number of significantly up- and down-regulated genes (EdgeR FDR < 0.1%) as a function of [THg]_intra_ (**B**). Venn diagrams showing the number of up- (red, upper numbers) and down- (green, lower numbers) significantly regulated genes in *C. reinhardtii* after 2 h exposure to IHg (**C**) or MeHg (**D**) (EdgeR, FDR < 0.1%).

Bioavailability of IHg and MeHg in the different treatments was then determined by measuring intracellular [THg]_intra_ and whole cell [THg]_intra+ads_ concentrations. At 10^−11^ and 10^−10^ M IHg, [THg]_intra_ represented 16 ± 1% of [THg]_intra+ads_ (Fig. 1A, Table S1), suggesting that the most part of Hg was loosely bound to the algal surface and extractable by molecules with strong affinity for Hg (i.e. cysteine). This ratio at all tested concentrations of MeHg was about 3.3× larger (53 ± 12%; Fig. 1A, Table S1), suggesting different fate for IHg and MeHg forms in *C. reinhardtii*. Indeed, no measurable demethylation of MeHg in the media or during uptake in *C. reinhardtii* was found. In fact, 100% of all [THg]_water_ were in the form of MeHg in MeHg treatments. Similarly, 100% of [THg]_intra_ were in the form of MeHg except at 10^−11^ M MeHg concentration where MeHg represented 30% of [THg]_intra_ due to the IHg background concentration in the algae. Furthermore, [THg]_intra_ increased linearly with the initial Hg concentration in exposure medium [THg]_water_ (IHg: n = 3, R^2^ = 0.996 ± 0.101, p = 0.03; MeHg: n = 4, R^2^ = 0.941 ± 0.218, p = 0.02; Fig. 1A, Table S1). At comparable exposure concentrations of 10^−11^ and 10^−8^ M IHg and MeHg, [THg]_intra_ was respectively 27× and 3× higher for MeHg exposure than for IHg exposure. These observations are in line with the results of field studies showing a 2× to 4× higher bioaccumulation of MeHg than IHg^6, 9^. However, they differ from the comparable intracellular contents of ^199^IHg and ^201^MeHg found in the 48 h exposure to their mixtures in the range 10^−12^–10^−9^ M^17^. The shorter exposure duration used here and the different exposure medium that affect speciation, and so Hg bioavailability, may explain this dissimilarity. Indeed, it is well established that speciation affects uptake, although Hg uptake pathways are still unclear^12^. Simple passive diffusion was proposed for small neutral lipophilic complexes (i.e. HgCl_2_^0^, and MeHgCl^0^) while uptake by carrier-mediated transport has also been demonstrated for other complexes^18–22^.

### Global changes in gene expression in response to IHg and MeHg

The number of reads ranged from 18.7 to 22.6·10^6^, with an average mapping of 86.2% (Table S2). Altogether, 8461 genes were dysregulated by Hg treatments, representing 47.7% of the total number of genes in *C. reinhardtii* genome. The numbers of down-regulated genes for both IHg and MeHg were larger than the up-regulated genes, except at 10^−8^ M MeHg (Fig. 1B, Tables S2 and S3). The number of dysregulated genes increased at higher [THg]_intra_ for both IHg and MeHg exposure. Furthermore, for comparable [THg]_intra_, MeHg induced dysregulation of a larger number of genes than IHg showing that MeHg induced a stronger molecular response in the cell. For example, at 1 amol_THg_·cell^−1^, 4.4× more genes were up-regulated by MeHg than IHg (Fig. 1B). Moreover, 15.7% of dysregulated genes had a fold change >4 or <−4 at 10^−8^ M MeHg concentration, while this percentage represented 0.4 to 6.7%, for 10^−11^ M IHg to 10^−9^ M MeHg, suggesting that the exposure to the higher concentration of MeHg resulted in a stronger molecular impact than other treatments. In addition, dysregulated genes specific to IHg represented only 4% (133) of all IHg regulated genes (3038), whereas 65% (5423) of MeHg affected genes (8328) were specific to MeHg (Fig. 1C,D). In summary, for comparable exposure concentration, greater uptake of MeHg than IHg was observed and for equivalent [THg]_intra_ a larger number of dysregulated genes for MeHg than IHg exposure were found. These observations support a different fate at the subcellular level of IHg and MeHg at similar exposure concentration.

### Metabolic pathways involved in *C. reinhardtii* transcriptomic and physiological response to IHg and MeHg

**Figure 2:**
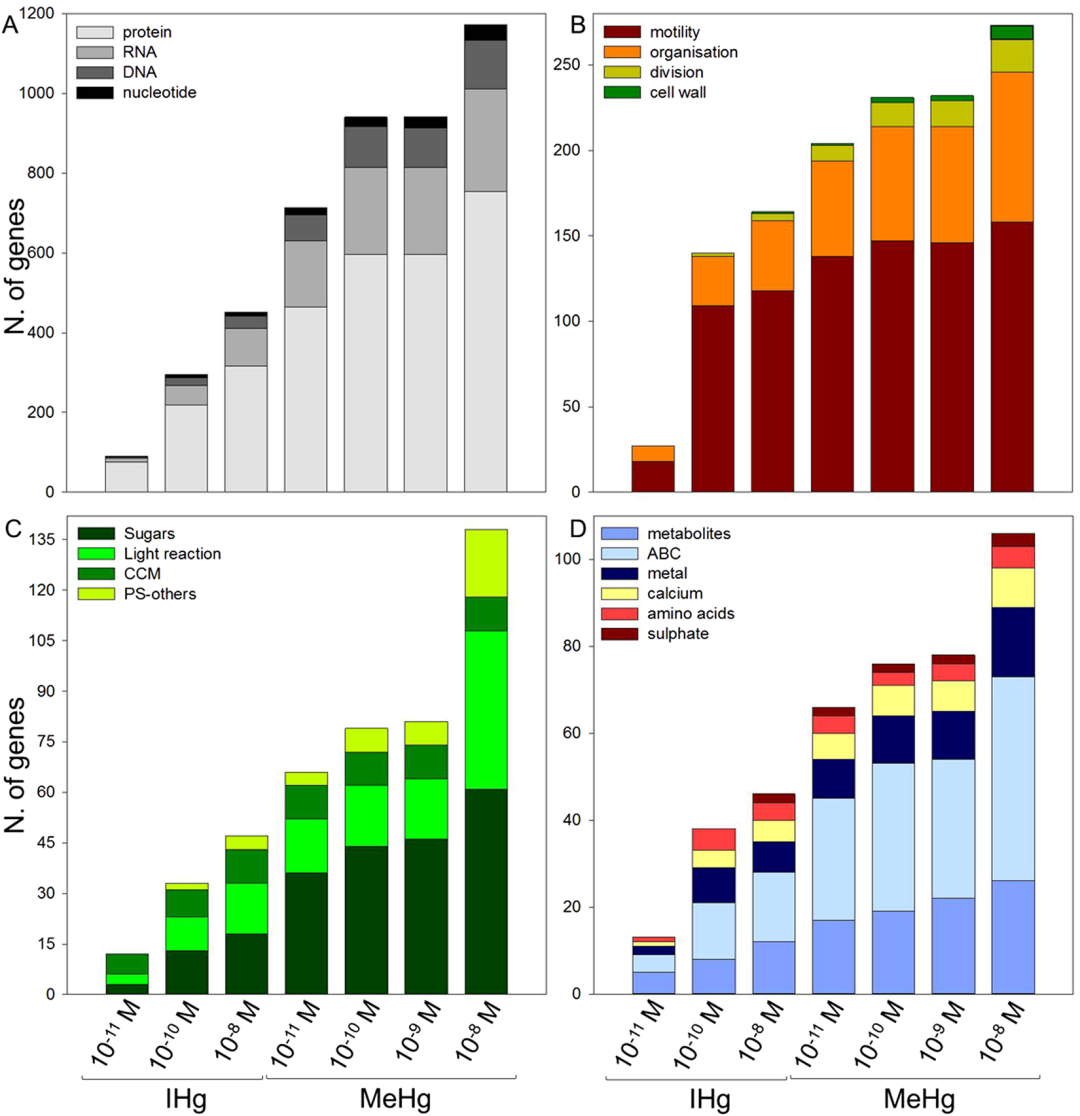
Number of genes significantly dysregulated in *C. reinhardtii* after 2 h exposure to increasing concentrations of IHg or MeHg (MapMan) in the categories involved in gene expression (**A**), cell processes (**B**), energy metabolism (**C**) and transport (**D**).

Although the “intensity” of the response was stronger at MeHg exposure, gene ontology (GO) of the differentially dysregulated genes showed that similar biological pathways were altered by both IHg and MeHg. These are pathways involved in regulation of gene expression (nucleotide to protein synthesis, signaling), cell processes (motility, division, development), energy metabolism (photosynthesis, sugar metabolism), lipid metabolism, amino acid metabolism, stress and transport, providing indications on cellular targets in *C. reinhardtii* (Table 1, Fig. 2, Figures S2 and S3, Tables S3 and S4).

**Table 1:** Number of significantly dysregulated genes in metabolic pathways (MapMan) in *C. reinhardtii* after 2 h exposure to increasing concentrations of IHg and MeHg (numbers in bold show categories with high dysregulation according to Wilcoxon test, *p* < 0.05).

Metabolic Pathways	Number of genes						
	IHg concentration (M)			MeHg concentration (M)			
	10^−11^	10^−10^	10^−8^	10^−11^	10^−10^	10^−9^	10^−8^
Amino acid metabolism	1	**10**	**17**	31	45	45	69
Cell processes	68	**455**	**505**	**611**	**668**	**665**	**746**
Energy metabolism	**23**	**60**	**83**	**110**	**125**	**127**	**250**
Gene expression	90	**295**	**452**	**714**	**941**	**942**	**1173**
Lipid metabolism	2	**13**	**18**	**42**	59	62	**78**
Other metabolisms	9	32	53	83	123	130	158
RedOx	0	7	9	18	24	24	34
Signaling	7	24	38	54	**66**	**68**	**87**
Stress	5	11	14	24	34	39	43
Transport	24	**70**	**87**	**141**	**165**	**168**	**218**

The number of dysregulated genes in all these categories increased from 2.8× to 17× for exposure to IHg from 10^−11^ to 10^−8^ M, and from 1.2× to 2.3× for exposure to MeHg from 10^−11^ to 10^−8^ M. The increasing number of dysregulated genes in the different categories was congruent with the increase of [THg]_intra_ measured for IHg or MeHg exposure and highlights the tolerance response of the microalga to tolerate increasing [THg]_intra_ (Figure S4). Interestingly, for similar [THg]_intra_, exposure to MeHg dysregulated 1.3× to 4.1× more genes in these categories than IHg exposures (Figure S4), supporting a greater impact on the cellular machinery of MeHg. Furthermore, these observations support a different subcellular fate for MeHg and IHg. The main dysregulated categories and the clues they give on subcellular targets of IHg and MeHg are discussed below.

#### Cell processes: motility, organization, division, and cell wall

Dysregulated genes involved in cellular processes, notably the cell motility and cell organization categories, were among the most strongly affected during exposure to both IHg and MeHg (Table 1, Table S4). Genes involved in the cell motility showed a strong response at all concentrations. More specifically, 10 genes coding for flagellar associated proteins were already strongly down-regulated at 10^−11^ M IHg and the number of regulated genes further increased in the other treatments (27 and 36 down-regulated genes for 10^−10^ and 10^−8^ M IHg, respectively, and up to 44 down-regulated and 4 up-regulated genes at 10^−8^ M MeHg). The regulation of the expression of genes related to flagella supports a possible impact of Hg on *C. reinhardtii* motility, as reported for other metals and MeHg in microalgae^12, 23^. Here, several genes involved in the cell division were also dysregulated. In particular, the gene coding for HORMA Mitotic spindle assembly checkpoint protein was down-regulated in 10^−10^ and 10^−9^ M MeHg and 10^−10^ and 10^−8^ M IHg treatments while other transcripts in this category were up-regulated. Genes involved in the cell wall metabolism and lignin biosynthesis (phenylpropanoids), notably glycoproteins (e.g. Cre16.g668850.t1.3, hydroxyproline-rich glycoprotein, log_2_FC −0.79, FDR 5.61·10^−7^) and expansin (Cre08.g381600.t1.2, expansin A24, log_2_FC 2.71, FDR 8.88·10^−5^) were only up-regulated at 10^−8^ M MeHg concentration. Similarly, expansin was strongly up-regulated by salt stress subsequently inducing the formation of palmelloids in *C. reinhardtii* and therefore it has been hypothesized that it is involved in the extension of cell walls during cell division to tolerate stress^24^. Here, genes involved in the cell organization were also strongly dysregulated. For example, the expression of fibrillin (e.g. Cre14.g618050.t1.2, plastid-lipid associated protein PAP/fibrillin) was up-regulated in all treatments except at the 10^−11^ M IHg concentration. Fibrillin is part of the plastoglobule proteome^25^, suggesting an impact of IHg and MeHg exposure on the synthesis of reserve lipid bodies. Overall, the results demonstrate that genes involved in the development and motility of *C. reinhardtii* were affected by IHg and MeHg at all the concentrations tested.

#### Energy metabolism

Genes involved in the carbon concentrating mechanism (CCM) were strongly dysregulated in *C. reinhardtii* by both IHg and MeHg (Tables S3 and S4), which is in agreement with previous studies suggesting that carbon acquisition could be impacted by MeHg in *C. reindhardtii* ^12, 26^. For instance, LCIB, a gene involved in inorganic carbon (C) accumulation in the chloroplast^27^, was strongly up-regulated (Cre04.g223250.t1.3, log_2_FC 2.19 and FDR 5.12·10^−4^ for 10^−8^ M IHg, log_2_FC 2.68 and FDR 1.87·10^−6^ for 10^−11^ M MeHg, log_2_FC 3.55 and FDR 1.50·10^−13^ for 10^−10^ M MeHg, log_2_FC 3.66 and FDR 1.30·10^−14^ for 10^−9^ M MeHg, 4.37 and FDR 1.50·10^−23^ for 10^−8^ M MeHg). In addition, IHg and MeHg exposure also influenced genes involved in the tricarboxylic acid (TCA) cycle. This metabolism occurs in the mitochondria, supporting evidence that both Hg forms affected mitochondrial activity and therefore the whole energy metabolism.

At the physiological level, chlorophyll a content ([chl *a*]) was increased at 10^−10^ M IHg *vs* control while photosynthesis efficiency was increased for 10^−11^ and 10^−10^ M IHg and in all MeHg exposure concentrations *vs* control (Table 2). This increase followed a “bell shape” with a higher increase for intermediate concentrations of exposure, typical of a hormesis effect, resulting from overcompensating for a moderate stress^28^. Along the same lines, a recent study reported a hormetic effect on photosynthesis efficiency in *C. reinhardtii* at 10^−11^ and 10^−10^ M MeHg^12^. Interestingly, it was found that phototrophic bacteria growth rates increased with increasing IHg concentrations suggesting that Hg could fulfil a physiological function^29^. However here, at higher [THg]_water_, [chl *a*] tended to decrease (Table 2) in line with expected toxicity pathways of Hg^6, 7^. Nevertheless, transcriptomics and physiological analysis revealed here that *C. reinhardtii* was able to cope with the tested Hg concentrations and developed an efficient tolerance response.

**Table 2:** Effects of IHg and MeHg on physiological endpoints in *C. reinhardtii* after 2 h exposure (bold characters indicate significantly different result from the control; t-test *p*-value < 0.05).

	Treatment	Cells with affected membrane permeability (%)	Cells with increased intracellular ROS (%)	[chl *a*] (% of control)	F_v_/F_m_ (% of control)
IHg	Control	17.7 ± 3.4	12.0 ± 1.5	100.0 ± 0.6	100.0 ± 1.7
	10^−11^ M	14.9 ± 0.1	11.2 ± 1.3	106.4 ± 5.9	**107.2 ± 0.2**
	10^−10^ M	16.8 ± 2.6	11.6 ± 3.5	**110.5 ± 5.6**	**110.8 ± 0.9**
	10^−8^ M	21.5 ± 2.1	12.3 ± 1.6	99.5 ± 4.9	103.2 ± 0.6
MeHg	Control	17.7 ± 3.4	12.0 ± 1.5	100.0 ± 11.2	100.0 ± 1.6
	10^−11^ M	24.6 ± 6.4	14.9 ± 0.4	95.0 ± 1.0	**123.0 ± 1.8**
	10^−10^ M	24.4 ± 3.9	**16.3 ± 2.3**	101.7 ± 3.1	**125.0 ± 0.4**
	10^−9^ M	23.6 ± 1.6	15.0 ± 1.0	93.4 ± 18.9	**130.3 ± 1.9**
	10^−8^ M	18.5 ± 3.3	14.9 ± 0.9	78.8 ± 15.1	**115.6 ± 1.5**

#### Reduction–oxidation reaction (RedOx) homeostasis

IHg and MeHg impacted the expression of genes involved in RedOx homeostasis such as thioredoxin (both up- and down- regulated by IHg and MeHg), peroxiredoxin (4 and 2 genes up-regulated at 10^−8^ M MeHg and IHg, respectively), and cytochrome p450 (1 to 6 and 11 to 15 up-regulated genes in the case of IHg and MeHg, respectively). Glutathione peroxidase were also up-regulated at 10^−8^ M MeHg (Cre03.g197750.t1.2, GPX3, log_2_FC 1.41, and FDR 1.54·10^−7^), and 10^−8^ M IHg concentration (Cre10.g458450, GPX5, log_2_FC 1.05, and FDR 3, 15·10^−4^). These observations point to an enhancement of reactive oxygen species (ROS) production induced by MeHg and IHg, in accordance with expected toxicity, and a subsequent modification of the RedOx homeostasis network in *C. reinhardtii* ^6, 7^. For example, thioredoxin are key molecules in the response of plants to oxidative stress induced by toxic metals^30–32^. On the other hand, at the physiological level, a significant increase of the intracellular ROS was only observed at 10^−10^ M MeHg but there was no impact for all IHg and MeHg treatments on membrane permeability, which can result from ROS-dependent lipid peroxidation (Table 2). Oxidative stress occurs from imbalance in RedOx homeostasis in cells which results in an overproduction of ROS. Strong ROS production is expected for soft Lewis acids such as Hg^2+^ and CH_3_Hg^+^ and when photosynthesis and mitochondria are impacted as suggested by the genes dysregulated here (see the subsection *Energy metabolism* above). Here, RedOx homeostasis seems to be more strongly influenced by MeHg than IHg at the gene level, similar to all the other categories. However, the algal cells limit ROS enhancement through an efficient antioxidant response observed at the gene level, which results in an absence of oxidative stress (except at 10^−10^ M MeHg) and agrees with observations previously reported for MeHg in *C. reinhardtii* ^12^.

#### Amino acid, lipid, and other metabolisms

IHg and MeHg exposure resulted in the dysregulation of genes involved in the metabolism of the thiol-related amino acids -glutamate, cysteine, and methionine- as well as biotin (acetyl-coenzyme A [CoA] cofactor) (Table 1, Tables S3 and S4). Biotin -a cofactor of acetyl CoA involved in sulfur (S) metabolism- was significantly enriched in response to MeHg (e.g. Cre17.g733650.t1.3, biotin F, log_2_FC 0.95 and FDR 3.53·10^−4^, log_2_FC 1.02 and FDR 8.64·10^−5^, log_2_FC 1.01 and FDR 1.03·10^−4^, for 10^−11^, 10^−10^ and 10^−9^ M MeHg, respectively). Increased biosynthesis of biotin was observed in *C. reinhardtii* under conditions of S-deprivation^33^. The data presented here supports dysregulation of S metabolism in the presence of MeHg. S could be useful for detoxifying sequestration of IHg and MeHg as thiol groups have high affinity to Hg. For example, S-containing amino acids (i.e. methionine and cysteine) and the subsequently biosynthesized S-rich peptides, phytochelatins (PC) and glutathione (GSH) can act as chelating molecules^34, 35^ and could here be involved in a mechanism of tolerance to MeHg in *C. reinhardtii*.

#### Transport

**Figure 3:**
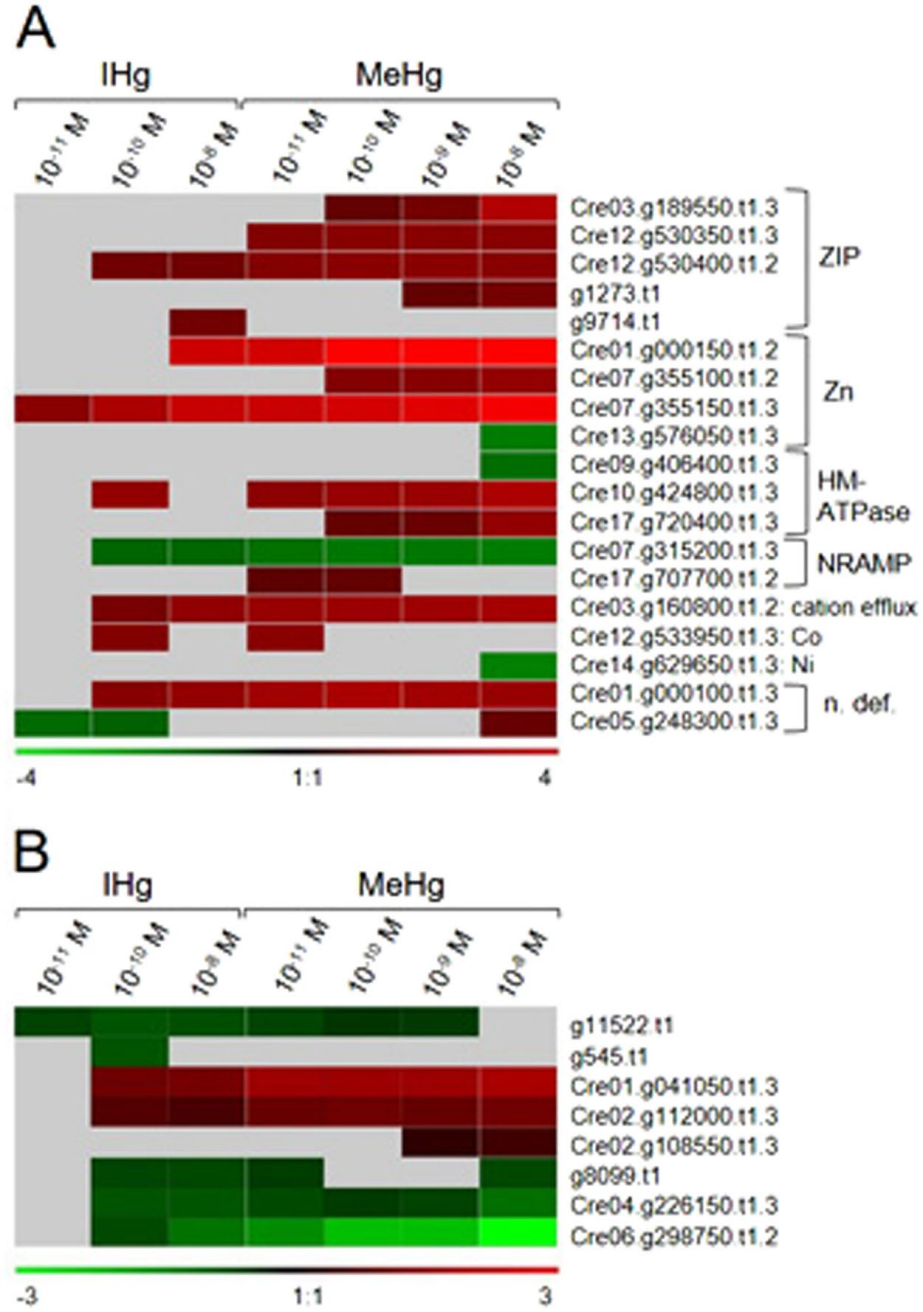
Gene expression regulation of 19 metal (**A**) and 8 amino acid (**B**) transporters significantly dysregulated in *C. reinhardtii* exposed 2 h to IHg or MeHg (ZIP = zinc-, iron-regulated transporter family; Zn = zinc transporter precursors; HM-ATPase = heavy metal ATPase; NRAMP = natural resistance-associated macrophage protein metal ion transporter family protein; Co = cobalt ion transmembrane transporter; Ni = high-affinity nickel-transport family protein; n. def. = no definition) (Phytozome V9.0).

Since the effects of contaminants depend on the intracellular concentration and their fate, the understanding of the uptake pathways and corresponding gene expression levels are of the highest interest. Genes involved in transporting metabolites, adenosine triphosphate (ATP) Binding Cassette (ABC) transporters, and metal transporters were dysregulated in all Hg treatments and similarly as in other categories, the number of dysregulated genes increased with [THg]_intra_ and was higher for MeHg than IHg (Table 1, Fig. 3). It is therefore tempting to hypothesize that several of these transporters are involved in Hg tolerance/detoxification mechanisms. Among the dysregulated ABC transporters, the multidrug resistance-associated protein 2 (MRP2), which was dysregulated in response to both Hg species here, has been identified as central in the transport of metals (*e.g*. cadmium; Cd) to the vacuole for detoxification^36^. Similarly, here these transporters could be involved in Hg vacuole detoxification, a well-known tolerance mechanism for toxic metals in primary producers^34^. Furthermore, genes involved in calcium (Ca^2+^) and S transport were significantly dysregulated by both IHg and MeHg. These regulations suggest an increase of intracellular ROS production. Indeed, Ca^2+^ has a major function as intracellular messenger, and act in concert with ROS signaling pathways in various cell processes such as growth and phototaxis in *C. reinhardtii* ^37, 38^. Significantly, an increase of ROS production causes an increase of [Ca^2+^]_intra_ and vice-versa^39^. Similarly, S transport is linked to the thiol pool that can play a role in Hg chelation and vacuolar compartmentation as well as in ROS scavenging by stimulating increases in glutathione biosynthesis^40^. This hypothesis agrees with the induction of genes involved in RedOx homeostasis by IHg and MeHg exposure in *C. reinhardtii* discussed in the section concerning *RedOx homeostasis* (see above).

Several zinc (Zn)-regulated transporters iron (Fe)-regulated transporter-like Proteins (ZIP) transporters of Fe and Zn as well as Zn transporters were also up-regulated by IHg and MeHg (Fig. 3A,B). At the higher concentration of MeHg, the nickel (Ni) high-affinity transporter, a heavy metal ATPase, and a Zn transporter were down-regulated. These observations suggest impact of IHg and MeHg on the global nutrition of the microalgae, and are in line with what has been observed in bacteria and plants in which essential-metal transporters are considered as potential uptake pathways for Hg^21, 41^. Indeed, a competition between IHg and Cu uptake was observed in *Elodea nuttallii* as well as IHg and Zn uptake in bacteria^21, 41, 42^. In addition, a close chemical mimicry exists between cysteine-MeHg and methionine, the hypothesis being made that the mechanism of Hg uptake in bacteria could be through amino-acid transporters^43–45^. Overall, here both IHg and MeHg dysregulated transporters are involved in nutrition, metal homeostasis, and in amino acid transport, suggesting that Hg may enter cells and/or be detoxified by those transporters. Further studies, notably identification and functional characterization of specific transporters, are needed to unambiguously demonstrate the carrier-mediated transport of Hg.

#### Genes specific to IHg and MeHg exposures

Overall comparison of genes dysregulated by IHg and MeHg exposures showed an absence of specific expression response to IHg (Table S5). Nonetheless, further detailed analysis of the genes specifically dysregulated for a given concentration, revealed differences in the proportion of the genes responding specifically to IHg treatments in some of the metabolic pathways. For example, 33% and 67% of genes specifically down-regulated at 10^−11^ M IHg were involved in development and stress, whilst these percentages were only 9% and 8% at 10^−11^ M MeHg exposure (Table S5). Genes involved in secondary metabolism represented 12% or 2% of specifically up-regulated genes by 10^−11^ M IHg or MeHg respectively (Table S5). Similarly, bigger percentage (11% up and 20% down) of genes involved in the stress category specific at 10^−10^ M IHg treatment were found in comparison with the same exposure concentration for MeHg (2% up and 8% down) (Table S5). Analogical trends were found for genes involved in lipid metabolism and mitochondrial electron transport/ATP synthesis specifically down-regulated by 10^−8^ M IHg and MeHg. Other metabolic pathways were more represented in MeHg treatments, probably due to the bias of the higher number of genes responding to MeHg, resulting in very limited IHg-specific impacted biological pathways. To reduce this bias, we thus compared IHg and MeHg treatments showing a closer number of dysregulated genes, namely the highest IHg concentration (2836 genes) and the lowest MeHg concentration (4174 genes; Table S6). As before, none of the metabolic pathways was dysregulated specifically by IHg and genes dysregulated by MeHg were found in more categories than those dysregulated by IHg (29 and 17, respectively). Genes involved in energy metabolism and development appeared to represent a higher proportion of genes specifically dysregulated by 10^−8^ M IHg than by 10^−11^ M MeHg, while genes involved in lipid metabolism represent a higher proportion of genes specifically dysregulated by 10^−11^ M MeHg than by 10^−8^ M IHg.

Considering the complete set of significantly dysregulated genes by IHg or MeHg, 134 genes were found to be specifically dysregulated by IHg treatments; however 96 of these genes have an unidentified function (Table 3). Similarly from the 5493 genes specifically dysregulated by MeHg, 3569 had an unidentified function. Globally IHg dysregulated genes were attributed to 16 categories, while MeHg dysregulated genes were attributed to 34 categories and none of the metabolic pathway was specific to IHg. Further analysis revealed that only 19 common genes were significantly dysregulated specifically by the three IHg treatments, while 981 genes were unique to the four MeHg treatments and absent in responses to IHg (Table 3). These findings show a stronger and more specific response of *C. reinhardtii* to MeHg exposure. However, 63% of genes specifically responding to MeHg exposure have an unknown function, 19% are related to gene expression regulation and signaling, 4% to cell organization and motility, 4% to transport, 1% to amino acids metabolism, and 1% to lipid metabolism. Similarly, for IHg, 10 of 19 genes have an unknown function, the others being involved in gene regulation and signaling, transport, cell processes, and development.

**Table 3:** Number of significantly dysregulated genes in metabolic pathways (MapMan) specifically responding to IHg or MeHg (specific) and common to all tested concentrations (specific and common) in *C. reinhardtii* after 2 h exposure.

Metabolic pathways	Number of genes			
	Specific and common		Specific	
	IHg	MeHg	IHg	MeHg
Amino acid metabolism		12	1	66
Biodegradation of Xenobiotics		1		8
C1-metabolism		2		7
Cell processes	1	41	2	147
Cell wall				10
Co-factor and vitamine metabolism		4		22
Development	1	8	1	38
DNA		24	1	113
Fermentation				2
Gluconeogenesis/glyoxylate cycle		1		4
Glycolysis		3		14
Hormone metabolism		2	1	16
Lipid metabolism		11	1	74
Major CHO metabolism		5		22
Minor CHO metabolism		4		20
Metal handling				7
Miscellaneous		20	4	101
Mitochondrial electron transport/ATP synthesis		2		10
N-metabolism		1		14
Nucleotide metabolism		7		32
OPP				3
Polyamine metabolism				3
Photosynthesis		1	3	54
Protein	4	116	5	536
Redox		3	1	31
RNA	2	54	6	238
S-assimilation				3
Secondary metabolism		1	1	25
Signaling	1	14	2	66
Stress		7		30
TCA/org transformation		2		11
Tetrapyrrole synthesis		4	3	16
Transport	1	38	6	161
Unknown	10	606	96	3569
Total	20	994	134	5493

Therefore, specific genes responding to MeHg includes notably a higher proportion of transporters, nevertheless no clear conclusions can be drawn about possible difference between MeHg and IHg subcellular targets and tolerance responses in *C. reinhardtii* in terms of dysregulated categories of genes as similar responses are observed and specific responses include many unknown genes.

## Conclusion

**Figure 4:**
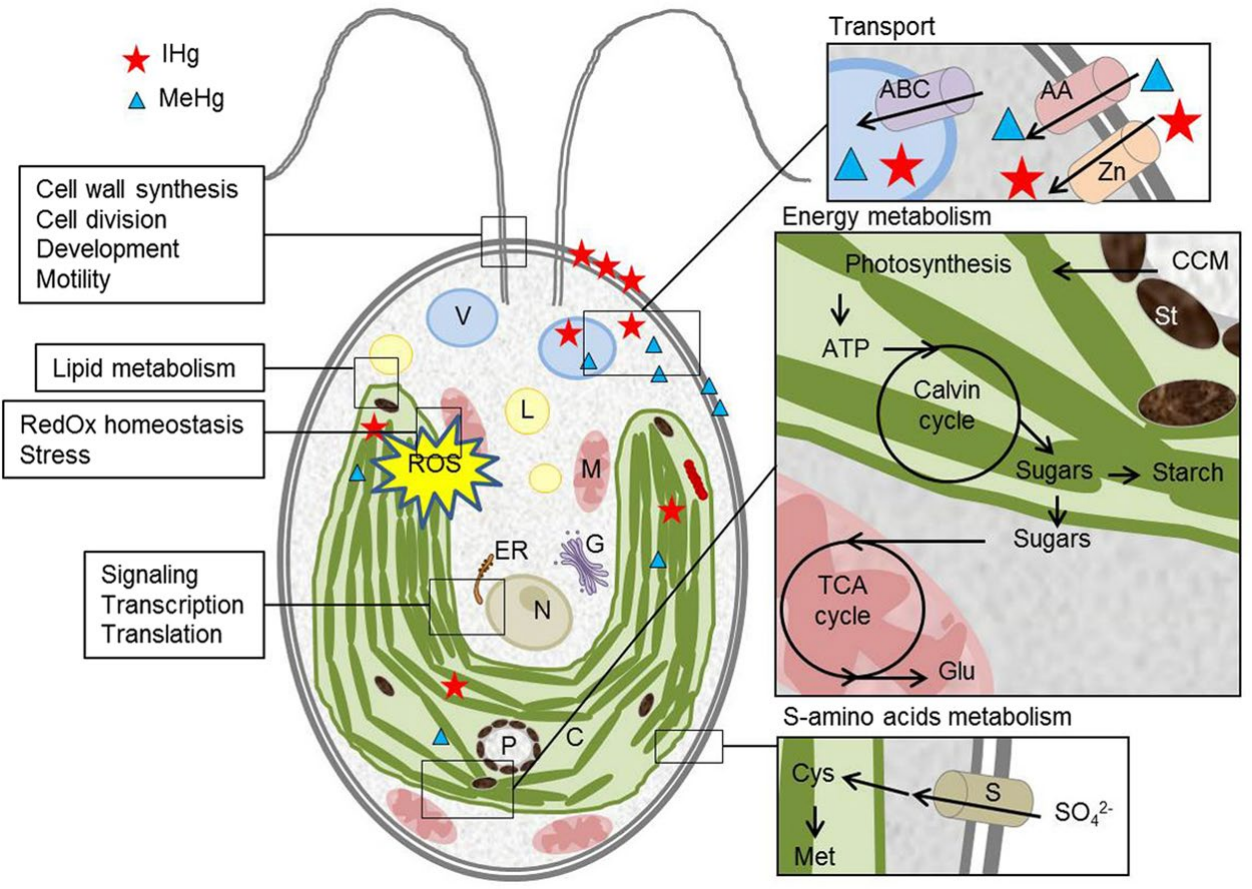
Schematic representation of cellular toxicity pathways and tolerance responses, as derived from uptake, transcriptome and physiological effects in *C. reinhardtii* exposed 2 h to IHg and MeHg (AA: amino acid transporter; ABC: ABC transporter; C: chloroplast; CCM: carbon concentrating mechanism; Cys: cysteine; L: lipid bodies; ER: endoplastic reticulum; G: golgi; Glu: glutamate; M: mitochondria; Met: methionine; N: nucleus; P: pyrenoid; S: S transporter; St: starch; V: vacuole; Zn: metal transporter).

This study highlighted the molecular-level responses of green microalga *C. reinhardtii*, a representative primary producer, to a broad range of environmental exposure concentrations of IHg or MeHg (Fig. 4). For similar [THg]_water_, exposure to MeHg resulted in a higher [THg]_intra_ in *C. reinhardtii* compared with exposure to IHg. The number of dysregulated genes increased with the intracellular concentrations for both IHg and MeHg exposure. However, for comparable intracellular concentrations [THg]_intra_, MeHg exposure dysregulated a larger number of genes than IHg exposure, supporting different subcellular fate of IHg and MeHg at similar concentrations in *C. reinhardtii*. Nevertheless, IHg and MeHg dysregulated similar categories of genes involved in cell processes, energy metabolism, photosystems, and RedOx homeostasis in agreement with expected toxicity. In addition, genes involved in cell motility, nutrition, and amino acids metabolism were dysregulated including during exposure to low environmental concentrations, revealing a broader impact on *C. reinhardtii* metabolism than expected based on physiological endpoints. Despite the strong gene response for both IHg and MeHg, the respective physiological responses were low. Only one treatment resulted in a significant increase of the cellular ROS and oxidative stress (10^−10^ M MeHg, Table 2), suggesting that the microalga could cope with the range of concentrations of IHg and MeHg tested here (10^−11^ to 10^−8^ M), at least for a short-exposure period. Overall, a detailed mechanistic understanding of differences in cellular handling of IHg and MeHg resulting in a stronger impact of MeHg were obtained from the analysis of the uptake and perturbations of the cell’s functions presented in this paper (Fig. 4). This knowledge is central for the development of integrated effect assessments and predicting the impact of toxicants on organisms.

## Methods

### Algal culture and exposure conditions

The unicellular green alga *Chlamydomonas reinhardtii* (wild type strain CPCC11) was obtained from the Canadian Phycological Culture Centre (CPCC, Department of Biology, University of Waterloo, Waterloo, ON, Canada). Cultures were grown in a specialized incubator (Multitron Infors HT, Bottmingen, Switzerland) at 20.2 ± 0.5 °C with a 24-h light cycle (3600 lux) and a constant 115 rpm rotary shaking. Cells were grown in a 4× diluted Tris-Acetate-Phosphate medium (Harris, 1989) containing tris-acetate, ammonium, phosphate, ethylenediaminetetraacetic acid (EDTA) and metals (B, Mn, Fe, Zn, Co, Mo, Cu) at pH 7.0.

For the exposures, cells were harvested in their middle exponential growth phase (62 h post inoculation) by centrifugation (10 min at 1300 *g*) and washed once with Hg free exposure medium (8.2·10^−4^ M CaCl_2_, 3.6·10^−4^ M MgSO_4_, 2.8·10^−4^ M NaHCO_3_, 1.0·10^−4^ M KH_2_PO_4_ and 5.0·10^−6^ M NH_4_NO_3_ at pH 6.9 ± 0.1). Subsequently cells were centrifuged again and re-suspended in the exposure medium. Cells were then exposed in the same incubator (see conditions above) at a cell density of 1·10^6^ cells·mL^−1^ for 2 h. This exposure duration was chosen based on toxicokinetics results for *C. reinhardtii* to both identify early response genes at the transcriptome level and reach measurable bioaccumulation^12^.

All materials were washed in 10% HNO_3_ followed by 10% HCl acid baths, thoroughly rinsed with ultrapure water (MilliQ Direct system, Merck Millipore, Darmstadt, Germany) and dried under a laminar flow hood. Material for culture and experiments, including media, were additionally autoclaved (1 bar, 121 °C, 20 min) to avoid microbial contamination.

### Hg bioaccumulation in *C. reinhardtii* and speciation

*C. reinhardtii* cells were exposed to 10^−11^, 10^−10^ or 10^−8^ M IHg using Hg(NO_3_) standard solution (Sigma-Aldrich, Buchs, Switzerland) or 10^−11^, 10^−10^, 10^−9^ or 10^−8^ M MeHg using MeHgCl standard solution (Alfa Aesar, Ward Hill, MA, USA). Cells exposed in the absence of Hg in the exposure medium were used as control. Exposure medium samples were preserved in the dark at 4 °C with 0.5% Suprapur® HCl (Merck, Darmstadt, Germany).

Speciation of IHg and MeHg in the exposure medium was computed using the Windermere Humic Aqueous Model (WHAM) model VII^46^. Its default database was updated for OH^−^, Cl^−^, SO_4_^2−^ and CO_3_^2−^ binding constants^47^.

At the end of the exposure, two aliquots (×3 replicates) of 50 mL were sub-sampled. After centrifugation, cells in the pellet were washed once with Hg-free exposure medium. The pellet of one of the aliquots was kept to measure the “whole cell” Hg concentration ([THg]_intra+ads_). The second aliquots were re-suspended in 10^−3^ M EDTA for IHg or 10^−3^ M EDTA + 10^−3^ M cysteine (Sigma-Aldrich, Buchs, Switzerland) for MeHg prepared in the exposure medium, to rinse adsorbed or loosely bound to the cell wall Hg to determine the intracellular Hg concentrations ([THg]_intra_)^48^. All pellets were freeze-dried (Beta 1–8 K, Christ, Germany) and kept in the dark until analysis.

Total Hg (THg = IHg + MeHg) concentration in algae was determined by atomic absorption spectrometry using the Advanced Hg Analyzer AMA 254 (Altec s.r.l., Czech Republic). Detection limit defined as 3× the standard deviation (s.d.) of 10 blank measurements was 0.05 ng_THg_. The accuracy of the measurements was checked by analyzing the certified reference material (CRM) MESS-3 (100 ± 0.1% recovery). Exposure medium samples were analyzed for initial THg concentrations with the MERX^®^ Automated Total Mercury Analytical System (Brooks Rand Instruments, Seattle, WA, USA). Detection limit was 0.03 ng_THg_·L^−1^. The accuracy of THg measurements was tested by analyzing the CRM ORMS-5 (116.0 ± 3.5% recovery). MeHg concentration was measured in exposure medium samples and in algae after mineralization 12 h at 60 °C with 30% HNO_3_ suprapur® (Merck, Darmstadt, Germany) with the instrument MERX^®^ Automated Methylmercury Analytical System (Brooks Rand Instruments, Seattle, WA, USA) following the United State of America Environmental Protection Agency (US EPA) Method 1630^49^ and manufacturer instructions. Detection limit was 0.01 ng_MeHg_·L^−1^. The accuracy of MeHg measurements was tested by analyzing the CRM TORT-2. Its recovery was 57.1 ± 5.3%.

### RNA-Seq and differential gene expression analysis

#### Total RNA extraction

At the end of the exposure, ~10^7^ cells were harvested by centrifugation (10 min at 1300 *g*) in triplicates and immediately frozen in liquid nitrogen and stored 1 night at −80 °C before total RNA extraction was conducted. For this, 0.5 mL of steel beads (0.50–0.75 mm, Retsch, Haan, Germany) and 1 mL TRI Reagent® (Sigma-Aldrich, Buchs, Switzerland) were added to the frozen algal pellet and the samples were shaken 30 s at 30 Hz (Mixer Mill MM 400, Retsch GmbH, Haan, Germany). Total RNA was then extracted following provider’s instructions. The amount and quality of RNA was confirmed by electrophoresis gel, measurement of total RNA concentration with Qubit® (Life Technologies Europe, Zug, Switzerland) and Agilent Bioanalyzer RNA 6000 Nano Kit (Agilent Technologies Inc., Santa Clara, CA, USA).

#### Library preparation and sequencing

Libraries were prepared following manufacturer’s protocols (Illumina, San Diego, CA, USA) and sequenced on an Illumina HiSeq. 2500 System (Illumina, San Diego, CA, USA), generating single-end reads of 100 bp. Data quality was controlled using the open source software FastQC (www.bioinformatics.babraham.ac.uk/projects/fastqc/).

#### Bioinformatics pipeline and differential gene expression analysis

Samples were aligned with TopHat2 to the Creiinhardtii 236 V.9.0 available at phytozome.org, and genes sequences and annotations were retrieved (ftp://ftp.jgi-psf.org/pub/compgen/phytozome/v9.0/Creinhardtii/annotation/). Reads were counted using the Python package HTSeq^50^. Data were deposited in the Gene Expression Omnibus database (GSE65109).

#### Differential gene expression analysis

Comparison of each IHg and MeHg conditions *vs*. Control generated fold change (FC) and false discovery rate adjusted *p*-values (FDR) was performed in the software CLC Main Workbench (Version 7, CLC bio, QIAGEN, Denmark) based on normalized counts and EdgeR package^51^. Significant differently expressed transcripts *vs*. control were defined with a threshold of false discovery rate (FDR) < 0.1% and of fold change log2 transformed (log_2_FC). Venn diagrams were realized with http://bioinformatics.psb.ugent.be/webtools/Venn/.

#### Functional annotation and enrichment analyses

Ontology term assignments were done using MapMan (Version 3.6.0RC1)^52, 53^. Wilcoxon test were used to identify most strongly dysregulated categories in each treatment (*p* < 0.05). Gene information was also retrieved using AlgeaPath and the Algal Functional Annotation Tool^54, 55^.

#### Reverse transcriptase quantitative Polymerase Chain Reaction (RT-qPCR)

To validate the RNAseq, RT-qPCR analysis was performed. Details can be found in the Supporting information.

### Physiological endpoints

The effects of IHg and MeHg on algal physiology were assessed by studying four endpoints for all the tested concentrations. The *oxidative stress* level of cells was assessed by the presence of intracellular ROS with the fluorescent probe Cell Rox Green Reagent® (Life Technologies Europe, Zug, Switzerland) added to a 1 mL subsample at a final concentration of 5·10^−6^ M. After 30 min incubation in the dark, the percentage of cells suffering from oxidative stress was assessed by flow cytometry (BD Accuri C6, BD Biosciences, Allschwil, Switzerland) with the detector FL1 (530 ± 15 nm) (positive control: cells exposed 1 h to 2·10^−4^ M 30% H_2_O_2_ (Suprapur®, Merck Millipore, Darmstadt, Germany). To assess the viability of the cells, *membrane integrity* was measured with propidium iodide (Sigma-Aldrich, Buchs, Switzerland), a fluorescent intercalating agent, added to the sample at a final concentration of 7·10^−6^ M and analyzed with the detector FL2 (585 ± 20 nm) of the flow cytometer (positive control: cells exposed 30 min to 1·10^−2^ M H_2_O_2_). The *efficiency of the photosynthesis* was assessed using Fast repetition rate fluorometry (FRRf) on 30 min dark adapted algae^56, 57^. The ratio of variable fluorescence (F_v_) over maximum fluorescence (F_m_) was measured indicating the maximum potential efficiency of photosystem II. The *chlorophyll a content* ([chl *a*]) was additionally determined with the Trilogy® Laboratory Fluorometer equipped with the chlorophyll *a in vivo* module (Turner Designs, Sunnyvale, CA, USA.).

### Data analyses

Data were tested for normality (Shapiro-Wilk, α = 0.01) and homoscedasticity (α = 0.01) and subsequently Student t-test was applied to compare treated *vs*. control samples. All statistical analyses and plots were computed using Sigma Plot© V12.5 (Systat Software, Inc., San Jose, CA, USA). Clusters and heatmaps were computed with Genesis v1.7.7 (Institute for Genomics and Bioinformatics, Graz University of Technology, Graz, Austria)^58^.

## Electronic supplementary material

Supplementary Information

Table S3

Table S4
