# Supplementary material for: Cellular toxicity pathways of inorganic and methyl mercury in the green microalga Chlamydomonas reinhardtii
Source: Sci Rep. 2017 Aug 14;7:8034. doi: 10.1038/s41598-017-08515-8 (PMC5556115; doi:10.1038/s41598-017-08515-8)
Supplement: Supplementary file 1 — Supplementary Information [file 41598_2017_8515_MOESM1_ESM.pdf]

Supporting information

Cellular toxicity pathways of inorganic and methyl mercury in the green microalga *Chlamydomonas reinhardtii*

IHg and MeHg impact in *Chlamydomonas reinhardtii*

Rebecca Beauvais-Flück, Vera I. Slaveykova, Claudia Cosio*

Department F.-A. Forel for environmental and aquatic sciences, Earth and Environmental Sciences, Faculty of Sciences, University of Geneva, 66, boulevard Carl-Vogt, 1211 Genève 4, Switzerland.

Quantitative Reverse Transcriptase-Polymerase Chain Reaction Analysis (RT-qPCR)

The expression analysis of 7 transcripts was performed using RT-qPCR (Figure S5). Total RNA was extracted as described in main text from control and exposed cells in biological triplicates. The M-MLV Reverse Transcriptase (Invitrogen™, Thermo Fisher Scientific Inc., Reinach, Switzerland) was used to synthesized complementary DNA from 1 µg DNAse-treated total RNA (DNase I, Fermentas™, Thermo Fisher Scientific Inc., Reinach, Switzerland; 30 min, 37° C). Technical duplicates of qPCR were performed with KAPA SYBR Fast Universal 2x qPCR Master Mix (KAPA Biosystems, Wilmington, USA) using an Eco Real-Time PCR System (Illumina Inc., San Diego, USA) following manufacturer's instructions and using specific primers (Table S7). The $2^{-\Delta\Delta Cq}$ approach [1] was used to calculate relative expression of the amplicons using the housekeeping rRNA 18S gene for normalization [2].

1 Livak, K.J. & Schmittgen, T.D., Analysis of relative gene expression data using real-time quantitative PCR and the 2(-Delta Delta C(T)) Method. *Methods* 25 (4), 402-408 (2001).

2 Vandesompele, J. *et al.*, Accurate normalization of real-time quantitative RT-PCR data by geometric averaging of multiple internal control genes. *Genome Biol* 3 (7), Research0034 (2002).

Table S1*:* Measured initial Hg water concentrations, intracellular (intra) Hg concentrations, adsorbed (ads) Hg concentrations in *C. reinhardtii* after 2h exposure to IHg or MeHg measured as THg and ratio of intracellular Hg content. Adsorbed Hg concentration ($[THg]_{ads}$) were calculated by subtracting $[THg]_{intra}$ from $[THg]_{intra+ads}$.

	$[THg]_{water}$ (M)	$[THg]_{intra}$ ($amol\cdot cell^{-1}$)	$[THg]_{ads}$ ($amol\cdot cell^{-1}$)	$[THg]_{intra}$ /$[THg]_{intra+ads}$
Control	$3.58\cdot 10^{-12}$	$(4.22 \pm 0.73)\cdot 10^{-2}$	$(2.50 \pm 0.06)\cdot 10^{-2}$	0.63
10^{-11} M IHg	$(1.47 \pm 0.04)\cdot 10^{-11}$	$(2.63 \pm 0.36)\cdot 10^{-3}$	$(1.33 \pm 0.28)\cdot 10^{-2}$	0.15
10^{-10} M IHg	$(7.06 \pm 0.18)\cdot 10^{-11}$	$(7.02 \pm 0.89)\cdot 10^{-3}$	$(3.61 \pm 0.64)\cdot 10^{-2}$	0.16
10^{-8} M IHg	$(4.09 \pm 0.11)\cdot 10^{-8}$	$(2.67 \pm 0.17)\cdot 10^{0}$	$(2.83 \pm 1.03)\cdot 10^{0}$	0.49
10^{-11} M MeHg	$(2.83 \pm 0.19)\cdot 10^{-11}$	$(7.01 \pm 3.43)\cdot 10^{-2}$	$(3.18 \pm 0.51)\cdot 10^{-2}$	0.69
10^{-10} M MeHg	$(3.57 \pm 0.32)\cdot 10^{-10}$	$(1.61 \pm 0.36)\cdot 10^{-1}$	$(1.38 \pm 0.63)\cdot 10^{-1}$	0.54
10^{-9} M MeHg	$(3.38 \pm 0.04)\cdot 10^{-9}$	$(9.63 \pm 1.50)\cdot 10^{-1}$	$(1.39 \pm 0.42)\cdot 10^{0}$	0.41
10^{-8} M MeHg	$(3.55 \pm 0.07)\cdot 10^{-8}$	$(7.57 \pm 2.20)\cdot 10^{0}$	$(8.43 \pm 1.34)\cdot 10^{0}$	0.47

Table S2: Differential gene expression analysis (EdgeR, FDR <0.1%) and mapping statistics (mean ± standard deviation, n = 3) resulting from the TopHat2 Alignment on Creinhardtii 236 (*Chlamydomonas reinhardtii* version 5.3.1).

Treatment	Number of reads ($\cdot 10^6$)	% mapped	Number of significant transcripts			
			up-regulated	down-regulated	Total (% of all transcripts)	% with $\log_2 FC \geq \|4\|$
Control	22.6 ± 3.0	84.7 ± 1.3				
10^{-11} M IHg	21.1 ± 5.0	86.9 ± 2.9	143	414	557 (3%)	0.4
10^{-10} M IHg	20.2 ± 1.9	86.9 ± 0.9	600	1391	1991 (11%)	1.2
10^{-8} M IHg	18.7 ± 2.6	86.4 ± 0.7	1054	1782	2836 (16%)	3.7
10^{-11} M MeHg	22.3 ± 1.0	86.2 ± 1.7	1882	2292	4174 (24%)	4.1
10^{-10} M MeHg	22.6 ± 3.0	84.3 ± 1.8	2652	2901	5553 (31%)	6.2
10^{-9} M MeHg	22.5 ± 1.8	84.6 ± 1.9	2627	2971	5598 (32%)	6.7
10^{-8} M MeHg	21.6 ± 1.7	89.7 ± 1.6	3408	3486	6894 (39%)	15.7

Table S3: Significantly up- and down-regulated genes in response to 2h exposure to $10^{-11, -10, -8}$ M IHg or $10^{-11, -10, -9, -8}$ M MeHg in *Chlamydomonas reinhardtii* (Gene ID, EdgeR log_2FC, FDR and Phytozome V9.0 description) (XLSX).

Table S4: MapMan enrichment analyses for the significantly dysregulated genes in *Chlamydomonas reinhardtii* in response to 2h exposure to $10^{-11, -10, -8}$ M IHg or $10^{-11, -10, -9, -8}$ M MeHg (Wilcoxon rank test, *p*-value < 0.05) (XLSX).

Table S5: Proportion (%) of genes in metabolic pathways (MapMan) specifically up-regulated (up) or down-regulated (down) by each concentration of IHg or MeHg in *C. reinhardtii* after 2h exposure.

	10^{-11} M				10^{-10} M				10^{-8} M			
	up		down		up		down		up		down	
**Metabolic pathways**	**IHg**	**MeHg**	**IHg**	**MeHg**	**IHg**	**MeHg**	**IHg**	**MeHg**	**IHg**	**MeHg**	**IHg**	**MeHg**
Amino acid metabolism	0.0	2.1	0.0	1.6	2.7	1.5	2.5	2.1	2.8	2.1	1.7	0.9
Biodegradation of Xenobiotics	0.0	0.5	0.0	2.5	0.0	0.1	0.0	4.3	2.8	2.7	1.7	4.4
C1-metabolism	0.0	0.5	0.0	0.4	0.0	0.7	2.5	0.3	0.0	0.6	1.7	0.4
Cell	6.3	6.2	0.0	0.7	5.4	8.3	0.0	1.0	0.0	0.2	0.0	1.1
Cell wall	0.0	0.0	0.0	0.0	0.0	0.1	0.0	0.0	0.0	0.6	0.0	0.0
Co-factor and vitamine metabolism	0.0	1.6	0.0	0.1	0.0	1.6	0.0	0.3	0.0	0.9	1.7	0.0
Development	6.3	3.2	33.3	8.8	5.4	2.8	12.5	12.4	26.8	10.9	18.6	13.7
DNA	0.0	9.1	0.0	0.1	2.7	10.6	0.0	0.3	0.0	0.2	0.0	0.1
Fermentation	0.0	0.0	0.0	0.3	0.0	0.0	0.0	0.7	1.4	1.5	0.0	0.9
Gluconeogenesis / glyoxylate cycle	0.0	0.0	0.0	0.7	0.0	0.0	0.0	2.0	4.2	9.2	1.7	2.1
Glycolysis	0.0	0.2	0.0	0.1	0.0	0.3	0.0	0.2	0.0	0.0	0.0	0.1
Hormone metabolism	0.0	1.1	0.0	0.9	2.7	1.0	0.0	1.2	0.0	0.2	3.4	1.1
Lipid metabolism	6.3	3.8	0.0	1.9	2.7	3.3	7.5	0.8	7.0	2.4	0.0	2.0
Major CHO metabolism	0.0	1.3	0.0	0.6	0.0	1.0	0.0	0.3	0.0	0.0	0.0	0.4
Metal handling	0.0	0.0	0.0	0.3	0.0	0.0	0.0	0.3	0.0	0.9	0.0	0.6
Micro RNA, natural antisense, etc.	0.0	0.2	0.0	0.7	0.0	0.0	0.0	1.5	1.4	0.8	1.7	0.9
Minor CHO metabolism	0.0	1.3	0.0	0.4	2.7	1.2	0.0	0.5	1.4	1.3	5.1	0.3
Misc	6.3	8.4	0.0	2.5	2.7	8.3	0.0	4.1	0.0	3.8	1.7	4.0
Mitochondrial electron transport / ATP synthesis	6.3	0.3	0.0	0.7	0.0	0.3	0.0	0.8	1.4	0.0	5.1	1.0
N-metabolism	0.0	0.2	0.0	1.2	0.0	0.7	2.5	1.3	0.0	1.5	1.7	1.0
Nucleotide metabolism	0.0	1.8	0.0	0.1	0.0	1.6	0.0	0.7	0.0	0.2	0.0	0.7
OPP	0.0	0.0	0.0	0.0	0.0	0.0	0.0	0.0	0.0	0.0	0.0	0.0
Polyamine metabolism	0.0	0.0	0.0	0.9	0.0	0.0	0.0	0.8	4.2	1.0	1.7	1.4
Protein	31.3	23.7	0.0	36.0	29.7	25.3	30.0	38.1	21.1	25.1	25.4	35.4
Photosynthesis (PS)	0.0	2.5	0.0	0.3	0.0	1.5	0.0	0.7	0.0	0.7	0.0	0.6
Redox	0.0	1.4	0.0	22.4	2.7	0.7	10.0	10.4	2.8	8.8	3.4	7.7
RNA	6.3	15.8	0.0	1.5	18.9	15.9	0.0	2.3	0.0	1.7	1.7	2.1
S-assimilation	0.0	0.0	0.0	0.9	0.0	0.1	0.0	0.8	0.0	2.5	0.0	1.0
Secondary metabolism	12.5	2.1	0.0	4.6	5.4	1.2	2.5	4.3	5.6	1.9	5.1	5.6
Signaling	6.3	2.7	0.0	2.9	2.7	2.7	10.0	2.6	11.3	5.4	5.1	4.6
Stress	18.8	1.9	66.7	7.8	10.8	1.9	20.0	7.8	5.6	9.6	11.9	8.3
TCA / org transformation	0.0	0.5	0.0	0.0	0.0	0.4	0.0	0.0	0.0	0.2	0.0	0.1
Tetrapyrrole synthesis	0.0	0.6	0.0	0.1	0.0	0.9	0.0	0.2	0.0	0.1	0.0	0.3
Transport	12.5	10.8	0.0	0.6	8.1	8.8	0.0	1.2	4.2	1.3	1.7	1.3

Table S6: Proportion (%) of genes according in metabolic pathways (MapMan) specifically up-regulated (up) or down-regulated (down) by10^{-8} M IHg and 10^{-11} M MeHg in *C. reinhardtii* after 2h exposure.

Metabolic pathways	Up		Down	
	IHg 10^{-8} M	MeHg 10^{-11} M	IHg 10^{-8} M	MeHg 10^{-11} M
Amino acid metabolism	7.5	1.7	2.2	4.6
Biodegradation of Xenobiotics	0.0	0.3	0.0	0.4
C1-metabolism	5.0	0.6	0.0	0.0
Cell	10.0	6.3	4.3	12.4
Cell wall	0.0	0.0	0.0	0.0
Co-factor and vitamine metabolism	0.0	1.7	0.0	0.4
Development	5.0	3.2	4.3	1.5
DNA	0.0	10.0	2.2	0.4
Fermentation	0.0	0.0	0.0	0.4
Gluconeogenesis / glyoxylate cycle	0.0	0.0	0.0	1.2
Glycolysis	0.0	0.3	0.0	0.8
Hormone metabolism	0.0	0.3	2.2	0.4
Lipid metabolism	0.0	3.7	0.0	4.2
Major CHO metabolism	0.0	1.1	0.0	1.5
Metal handling	0.0	0.0	0.0	0.4
Micro RNA, natural antisense etc	0.0	0.0	0.0	0.0
Minor CHO metabolism	0.0	0.6	0.0	0.8
Miscellanous	2.5	7.7	8.7	3.1
Mitochondrial electron transport / ATP synthesis	0.0	0.6	2.2	0.8
N-metabolism	0.0	0.0	0.0	0.8
Nucleotide metabolism	2.5	2.3	2.2	0.4
OPP	0.0	0.0	0.0	0.0
Polyamine metabolism	0.0	0.0	0.0	0.0
Protein	20.0	23.5	32.6	34.4
Photosynthesis (PS)	10.0	1.7	4.3	0.4
Redox	2.5	1.1	0.0	1.9
RNA	15.0	16.3	10.9	10.0
S-assimilation	0.0	0.0	0.0	0.0
Secondary metabolism	2.5	1.1	0.0	0.8
Signalling	5.0	1.7	4.3	5.4
Stress	2.5	2.3	0.0	1.2
TCA / org transformation	2.5	0.0	0.0	1.5
Tetrapyrrole synthesis	2.5	0.9	6.5	0.8
Transport	5.0	10.9	13.0	9.3

Table S7: Primers used for the RT-qPCR.

**ID**	**Forward primer (5' → 3')**	**Reverser Primer (5' → 3')**	**Description**
Cre02.g097800.t1.3	CTTGGTGGTCTGTGCAAGTG	TAGTTGACGTGGGACAGCAG	Multidrug resistance-associated protein 2
Cre03.g149300.t1.3	CACCCACATCTCCCAGTTCT	CTGCGTGGGAATCTTGTTCT	Methylthiotransferase
Cre04.g222750.t1.1	TCAAGCAGGTCATGTCCAAG	TCTGCAGGAAGTGGTTGACA	Low-CO_2-inducible chloroplast envelope protein
Cre04.g224650.t1.2	TCCAACCAAAGTCATCACCA	GGAGCTCTTAGCTGCCTCAA	Flagellar Associated importin alpha-like protein
Cre09.g410750.t1.2	TTTGAGCTGTCTTCCCTGCT	AGGTCCTCCCACTTGGTCTT	Nitrite reductase
Cre10.g446550.t1.2	CAAGCCTTCAAGGAGTTTGC	CTTCAATGACGTTCCGGATT	Vacuolar ATP synthase subunit F
Cre17.g711150.t1.2	AACCATTTCGACCCCTTCTC	CGCAATATCAGAGACCAGCA	*No available description*
rRNA 18S	GGAGGATTAGGGTTCGATT	CGCCCGGTATTGTTATTTAT	Ribosome 18S

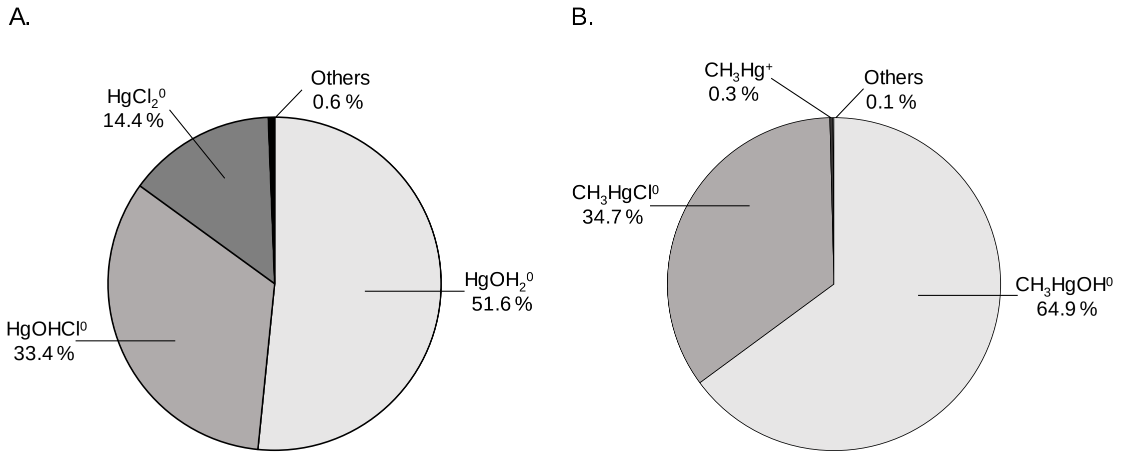

Figure S1: Speciation of IHg (A) and MeHg (CH_3Hg) (B) in the exposure medium. The values were constant between the different exposure concentrations (WHAM/ModelVII).

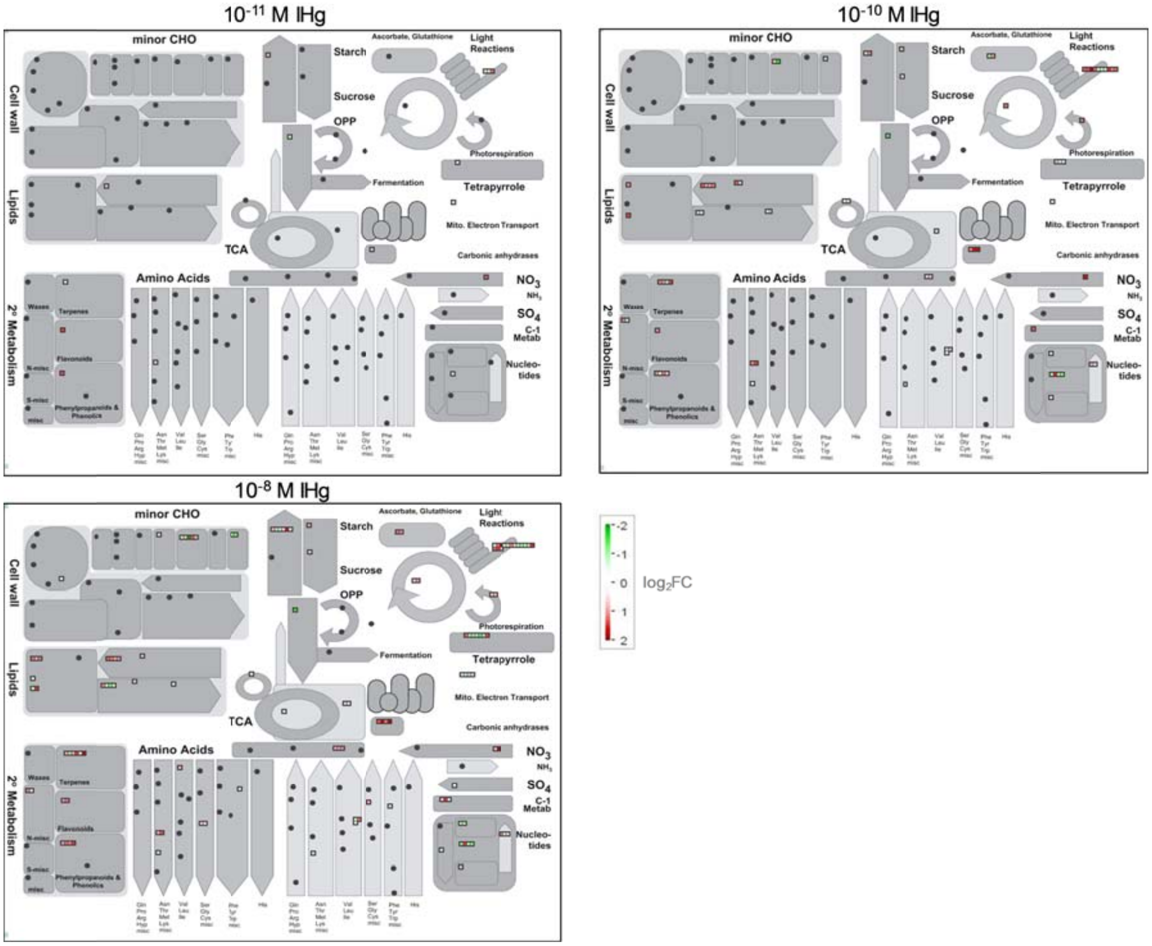

Figure S2: Metabolism overview (MapMan) for transcripts significantly dysregulated by IHg after 2h exposure of *C. reinhardtii*.

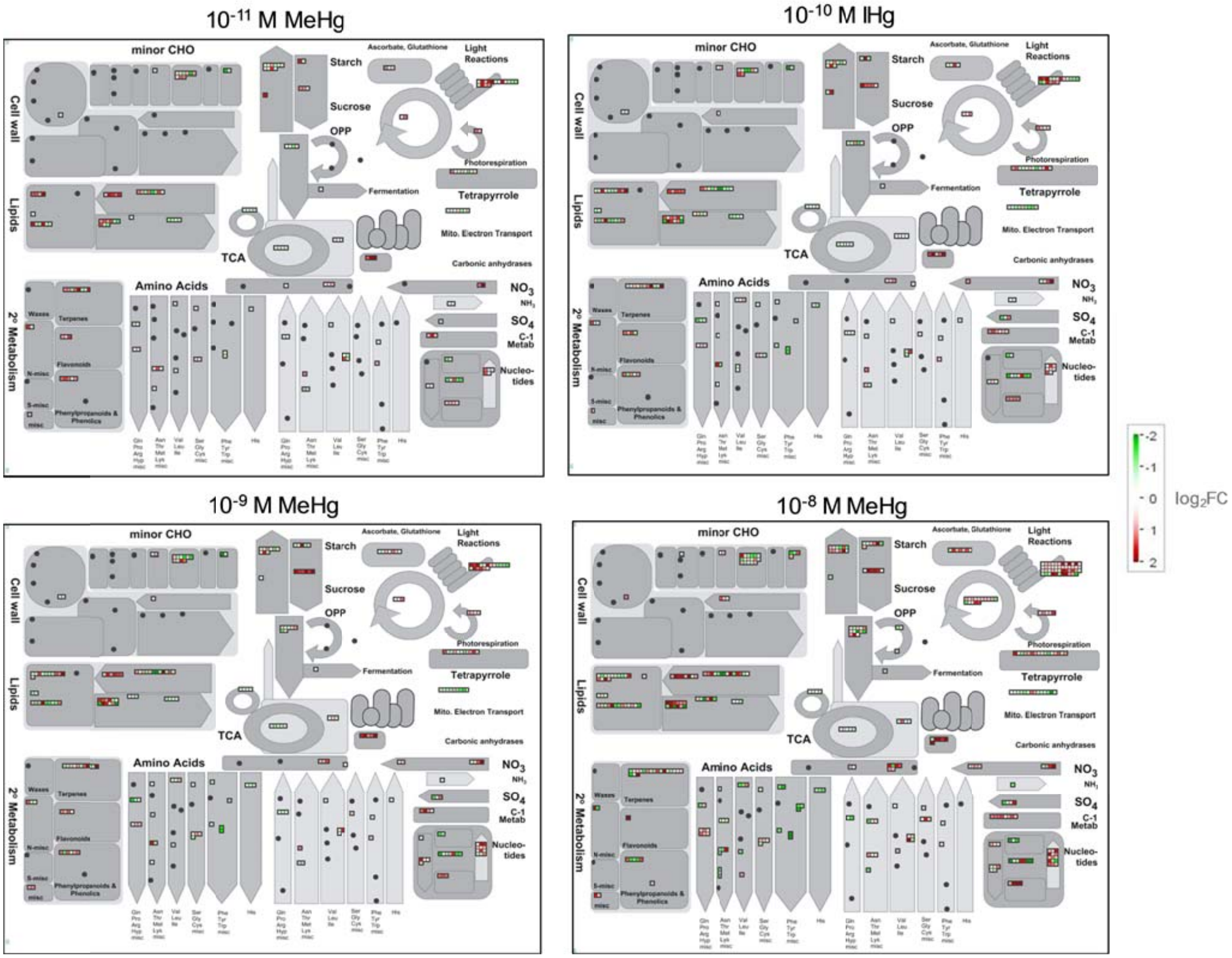

Figure S3: Metabolism overview (MapMan) for transcripts significantly dysregulated by MeHg after 2h exposure of *C. reinhardtii*.

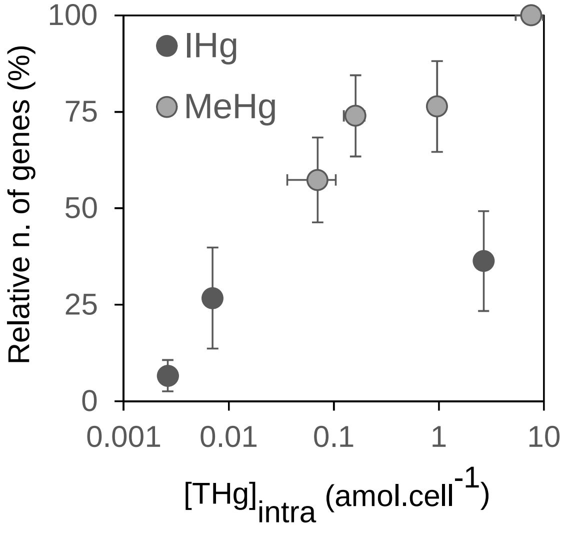

Figure S4: Relative number of genes significantly dysregulated in top categories of MapMan in *C. reinhardtii* after 2h exposure to IHg or MeHg as a function of internal total Hg concentration ($[THg]_{intra}$, mean ± sd, n = 3).

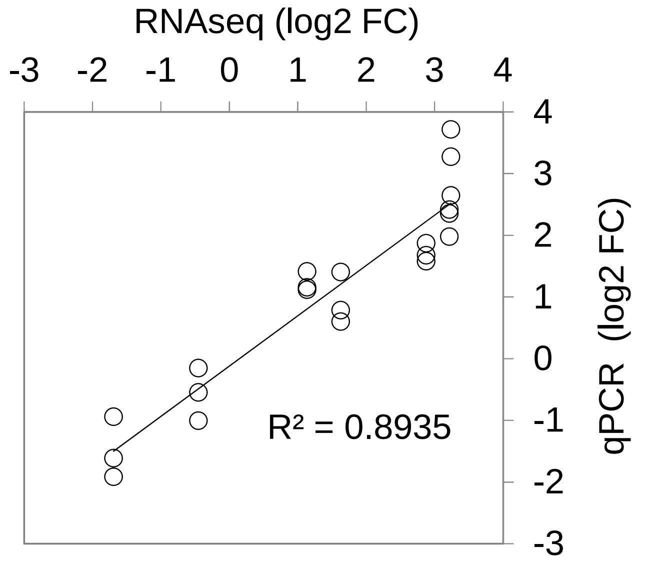

Figure S5: Linear regression between RNA-Seq and RT-qPCR fold change values (log_2FC). Seven genes found differentially expressed by RNA-Seq were analyzed by RT-qPCR (n= 3).
